# Stochastic Multi-Molecular Modeling Method of Organic-Modified Ceramics in Two-Photon Induced Photopolymerization

**DOI:** 10.3390/ma12233876

**Published:** 2019-11-24

**Authors:** Jieqiong Lin, Peng Liu, Xian Jing, Mingming Lu, Kaixuan Wang, Jie Sun

**Affiliations:** Key Laboratory of Micro/Nano and Ultra-Precision Manufacturing, School of Mechatronic Engineering, Changchun University of Technology, Changchun 130012, China; linjieqiong@ccut.edu.cn (J.L.); Liupeng@ccut.edu.cn (P.L.); lumm@ccut.edu.cn (M.L.); 2201801046@stu.ccut.edu.cn (K.W.); 2201801013@stu.ccut.edu.cn (J.S.)

**Keywords:** two-photon polymerization, stochastic multi-molecular modeling method, molecular dynamics analysis, organically modified ceramics, shrinkage

## Abstract

Organic-modified ceramics (Ormocer) are an outstanding class of hybrid materials due to the fact of their various excellent properties, and they have been successfully used in two-photon polymerization microfabrication fields. A series of functional devices has been fabricated and widely used in aerospace, information science, biomedicine, and other fields. However, quantization of intermolecular energy during the fabrication process is still a difficult problem. A stochastic multi-molecular modeling method is proposed in this paper. The detailed molecular-interaction energies during the photon polymerization of Ormocer were obtained by molecular dynamics analysis. The established molecular model was verified by comparing the simulated shrinkage results with commercial calibrated ones. This work is expected to provide a reference for optimizing the fabrication of organically modified ceramics and reducing photoresist shrinkage in two-photon polymerization.

## 1. Introduction

Ormocer is a kind of hybrid material which is synthesized by compounding organic matter and inorganic matter at the atomic or molecular scales [1,2,3]. Many excellent properties, including optical, mechanical, and biological, have been exhibited during their wide application in tissue engineering [4,5], micro-electromechanical systems (MEMS) [6,7], micro-optics [8,9], microfluidics [10,11], and metamaterials [12,13]. They are benefited by their co-existing inorganic and organic networks. Ormocer has become an important material in microfabrication fields, especially in two-photon induced photopolymerization (TPIP) [14,15,16]. Using this technology, sub-100 nm resolution and complex three-dimensional microstructures have been obtained. However, it is still difficult to dig deeply into this material’s reaction process at a micro/nano level and qualify the intermolecular energy transformation from experiments. Molecular dynamics simulation may provide a possible solution [17,18,19]. 

Many molecular dynamics modeling methods have been proposed to simulate the interaction among molecules at a micro level. Kisin et al. [20] used a fixed number of styrene and acrylonitrile units to build a polymer molecular model to simulate the adhesion of metal to polymer. Deetz et al. [21] established a model according to the molecular formulas of tetrahydroxysilane and trihydroxysilane monomers, and they used an iterative method to simulate the reaction of the molecular dynamics of the two polycondensates. Asche et al. [22] encapsulated the two monomers in a box with a 1:1 ratio and connected them one by one to simulate their polymerization process. However, there is still room for the improvement in molecular dynamics modeling accuracy. In this paper, a stochastic multi-molecular modeling method is proposed to simulate the photon polymerization process in TPIP and reveal the energy transform at a molecular level. A stochastic modeling method is proposed to establish monomers which can bring the modeling process closer to the actual polymerization of photoresist monomers. A large number of monomer structures were packaged together in order to ensure the accuracy of the cross-linking to the utmost extent, preventing the randomness of cross-linking due to the fact of an insufficient amount of photoresist which affects the quality of cross-linking and ultimately the accuracy of the simulation. Considering the accuracy of cross-linking and the computing performance of the workstations, 20, 25, and 30 monomers were selected for packaging. Lastly, the proposed modeling method was verified by the shrinkage property of the Ormocer. This work is expected to provide a reference for revealing the energy distribution among Ormocer molecules in TPIP fabrication processes and to predict the shrinkage of final fabricated structures.

## 2. Materials and Theory

### 2.1. Synthesis of Photoresist and Reaction Principle of Cross-Linking Polymerization

According to the different products generated by the initiator, the photoresist used in TPIP can be classified as cationic photoresist and free radical photoresist [23,24]. The photoresist used in this report was Ormocer which is a kind of free radical photoresist. It combines the properties of all three material classes (i.e., organic polymers, inorganic silica, and silicones) with resulting properties depending on the ratio and components used [25]. Three common Ormocer and their contents are listed in Table 1 [22,26].

**Figure 1:**
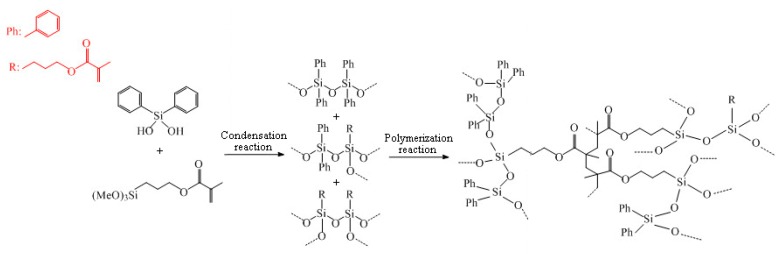
Schematic diagram of the two-step synthesis of Ormocer-II.

**Figure 2:**
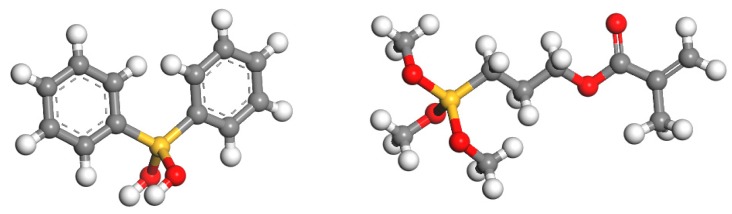
Precursor of Ormocer photoresist (white, gray, red, and yellow spheres represent hydrogen, carbon, oxygen, and silicon, respectively).

**Figure 3:**
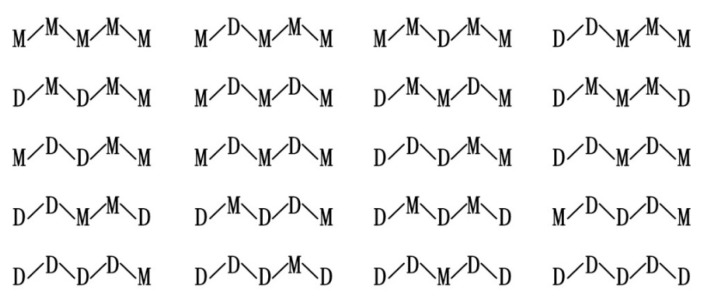
Twenty possible monomer molecules consisting of five Si–O skeletons. M stands for 3-methacryloxypropyl-trimethoxysilane (MEMO), D stands for diphenylsilanediol (DPD).

The photoresist used in this paper was formed by condensing two precursors of Ormocer-II in a ratio of 1:1. They were synthesized in a two-step process involving, firstly, a condensation reaction of inorganic precursor molecules and, secondly, a polymerization reaction, leading to a cross-linked organic–inorganic network [27]. The reaction process is shown in Figure 1 [19]. Two of the precursor structures are shown in Figure 2. Fessel et al. [19], according to small-angle X-ray scattering (SAXS) experiments, found that the size of these condensed Si–O skeleton structures is around 1 nm. Thus, the single molecule that satisfies this condition contains 4–6 Si atoms; these monomers are formed by the 1:1 condensation of two precursors in Ormocer-II. The model used here was a chain of five Si atoms, and all possible structures in the process of synthesizing monomers in the precursor are shown in Figure 3.

**Figure 4:**
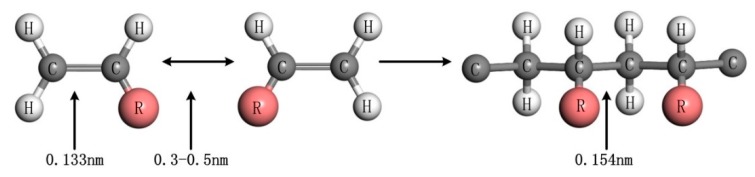
Molecular structural formula before and after cross-linking.

After the monomer molecular model was established, encapsulation was carried out to simulate the actual cross-linking reaction. Since the cross-linking reaction involves breaking the old bond and generating new bonds, this reaction was beyond the scope of the force field simulation. In order to overcome this limitation, this simulation used a manual cross-linking method to perform the molecular modeling of the model. Firstly, the model was simulated by dynamics and optimized by energy minimization, and then the optimized compound was cross-linked manually. The change in the molecular formula and the change in the distance between atoms before and after cross-linking are shown in Figure 4.

Manual cross-linking was adopted using the process in Reference [19]. Firstly, quench dynamic analysis was performed on the constructed photoresist model to achieve a stable structure; then, the distances among carbon atoms of different C=C in the model were measured with a measuring tool, and the shortest carbon atoms with a measured distance between 0.3 and 0.5 nm were manually cross-linked according to the principle in Figure 4 (firstly, the carbon atom with the shortest distance was connected by covalent bond, then the double bond was changed into a single bond, and finally the atom in the structure was balanced by an auto-update hydrogen button). After the cross-linking, a quench dynamics simulation was performed to achieve a stable structure, the distance measurements were continued, and the above operation was repeated until all eligible carbon atoms were cross-linked. At last, a quench dynamics simulation with 50 ps was performed to achieve a stable structure.

### 2.2. Theory of Mechanics

The force field used in this paper was the COMPASS (condensed-phase optimized molecular potentials for atomistic simulation studies) force field. The COMPASS force field can precisely simulate and predict the structure, conformation, vibrational frequency, and thermodynamic property of a single molecule or condensed matter to a large extent [28]. Its study objects can be organic small molecules, inorganic small molecules, polymers, etc. The COMPASS force field is more suitable for calculating the potentials of molecular structures during the TPIP process.

The bond stretching potentials can be obtained by:

(1) $$u_{b}\left(l\right)=D_{e}{\left\{1-\exp\left[-\alpha \left(l-l_{0}\right)\right]\right\}}^{2}$$

where *D_e_* and *α* are the coefficients related to the depth and width of the potential well, respectively, and *l*_0_ is the standard length of a bond [29].

Angle bending potentials can be obtained by:

(2) $$u_{\theta }\left(\theta \right)=\frac{1}{2}K_{\theta }{\left(\theta -\theta_{0}\right)}^{2}\left[1-\alpha \left(\theta -\theta_{0}\right)+\frac{7}{12}\alpha^{2}{\left(\theta -\theta_{0}\right)}^{2}+\cdots \right]$$

where *K_θ_* is the angle bending force coefficient and *θ*_0_ is the standard angle of the bond [30].

Torsional potentials represent the energy change caused by rotation around a bond which can be obtained by:

(3) $$u_{\omega }\left(\omega \right)=\frac{1}{2}\sum_{n}V_{n}\left[1+{\left(-1\right)}^{n+1}\cos n\omega \right]$$

where *V_n_* is the dihedral angular torsional potential coefficient which represents the energy barrier height and *n* is an integer representing the number of times that the minimum energy value appears when rotating 360° around the key; its value differs with the force field. *ω* is the vibrational frequency.

Out-of-plane bending potential is the potential of an atom when it oscillates up and down near the plane of the other three atoms which can be obtained by:

(4) $$u_{\chi }\left(\chi \right)=\frac{1}{2}K_{\chi }{\left(\chi -\chi_{0}\right)}^{2}$$

where *K_χ_* is the vibrational potential coefficient away from the plane and *χ* represents the angle or height of vibration away from the plane.

As the molecular movements of different forms are coupled, for example, the key expansion will cause the key to angle and dihedral angle changes, and vice versa. Cross-terms exist during energy transformation, and the relationships among them can be expressed by (Equation (5)).

The bond stretch–stretch potential can be obtained by:

(5) $$u\left(l_{1},l_{2}\right)=\frac{1}{2}K_{l_{1},l_{2}}\left(l_{1}-l_{1,0}\right)\left(l_{2}-l_{2,0}\right)$$

The bond stretch–bend potential can be obtained by:

(6) $$u\left(l_{1},l_{2},\theta \right)=\frac{1}{2}K_{l_{1},l_{2},\theta }\left[\left(l_{1}-l_{1,0}\right)+(l_{2}-l_{2,0})\right]\left(\theta -\theta_{0}\right)$$

where the bond angle *θ* is the included angle among two bonds.

The bond stretch–dihedral angular torsion can be obtained by:

(7) $$u\left(l,\omega \right)=\frac{1}{2}K_{l,\omega }\left(l-l_{0}\right)\left(1+\cos3\omega \right)$$

The bond angle bend–torsion potential can be obtained by:

(8) $$u\left(\theta ,\omega \right)=\frac{1}{2}K_{\theta ,\omega }\left(\theta -\theta_{0}\right)\left(1+\cos3\omega \right)$$

The bond angle bend–bond angle bend potential can be obtained by:

(9) $$u\left(\theta_{1},\theta_{2}\right)=\frac{1}{2}K_{\theta_{1},\theta_{2}}\left(\theta_{1}-\theta_{1,0}\right)\left(\theta_{2}-\theta_{2,0}\right)$$

Van der Waals potential is the potential energy among molecules. Dispersion force and repulsive force play important roles in this potential. The former is long range attraction, while the latter is short-range repulsive force. Van der Waals potential can be obtained by

(10) $$u_{dis}=-\frac{3\alpha^{4}h\omega }{4{\left(4\pi \varepsilon_{0}\right)}^{2}r^{6}}$$

where $\omega =\sqrt{k/m}$ is the angular frequency $\alpha =q^{2}/k$, where *k* is force coefficient and *q* is particle charge [31].

Non-bonding interactions among molecules will be more important when a molecule has a charge or a multipolar moment. The electrostatic interaction of electric charge *q*, dipole moment *μ*, and quadrupole moment *Q* between two different molecules *a* and *b* can be expressed according to the Coulomb law as follows:

(11) $$u^{\left(q,q\right)}\left(r\right)=\frac{q_{a}q_{b}}{r}$$

(12) $$u^{\left(q,\mu \right)}(r)=\frac{q_{a}\mu_{b}\cos\theta_{b}}{r^{2}}$$

(13) $$u^{\left(q,Q\right)}(r)=\frac{q_{a}Q_{b}(3\cos^{2}\theta_{b}-1)}{4r^{3}}$$

(14) $$u^{\left(\mu ,\mu \right)}(r)=-\frac{\mu_{a}\mu_{b}\left[2\cos\theta_{a}\cos\theta_{b}-\sin\theta_{a}\sin\theta_{b}\cos(\phi_{a}-\phi_{b})\right]}{r^{3}}$$

(15) $$u^{\left(\mu ,Q\right)}(r)=\frac{3\mu_{a}Q_{b}}{4r^{4}}\left[\begin{array}{c} \cos\theta_{a}\left(3\cos^{2}\theta_{b}-1\right)\\ -2\sin\theta_{a}\sin\theta_{b}\cos(\phi_{a}-\phi_{b}) \end{array}\right]$$

(16) $$u^{\left(Q,Q\right)}(r)=\frac{3Q_{a}Q_{b}}{16r^{5}}\left\{\begin{array}{c} 1-5\cos^{2}\theta_{a}-5\cos^{2}\theta_{b}-15\cos^{2}\theta_{a}\cos^{2}\theta_{b}\\ 2{\left[\sin\theta_{a}\sin\theta_{b}\cos(\phi_{a}-\phi_{b})-4\cos\theta_{a}\cos\theta_{b}\right]}^{2} \end{array}\right\}$$

where *u* represents the electrostatic interaction, *r* represents the distance between two molecules, *θ_a_* and *θ_b_* represent the plane angle of molecules a and *b*, respectively, and *ϕ_a_* and *ϕ_b_* represent the roll angles of molecules *a* and *b*, respectively.

This section introduces in detail the calculation methods for the parameters involved in the molecular dynamics simulation. In the process of simulation, these parameters were generated automatically.

## 3. Molecular Dynamics Simulation and Discussion

### 3.1. Establishment of Ormocer Photoresist Model

In the synthesis of Ormocer, the reaction of the precursor condensation to form monomer is random. In order to better reflect the randomness of the reaction, this paper used the Random Copolymer function in the Build Polymers module of MS 2017 software to establish the Ormocer monomer. MS 2017 is simulation software (Accelrys Materials Studio 3.0.) developed for researchers in the field of materials science that runs on a PC. It can help solve some of the problems in today’s chemistry and materials industries. The Random Copolymer dialog allows you to build random copolymer chains with various orientations, tacticities, and conditional probabilities. This method can completely simulate the generation process of a photoresist monomer for monomer modeling which makes the model more consistent with the actual structure of photoresist.

In order to make the simulation more consistent with the actual condensation reflection, this paper adopted a random method to establish monomer molecules and then encapsulated monomer molecules together. If the number of encapsulated molecules was too small, the randomness of the polymerization was too large and not convincing. If the number of packages was too large, the accuracy of the cross-linking and the speed of the calculation would be seriously affected. Based on the above two factors, we selected 20, 25, and 30 monomers as the research objects for three sets of simulations. The simulations in this paper only took 20 monomers as examples for modeling, and the model of the other two groups of structures is referred to in Supplementary Materials. According to our modeling principle, 20 randomly generated monomers were packaged together as our photoresist model.

### 3.2. Dynamic Simulation before Cross-Linking

**Figure 5:**
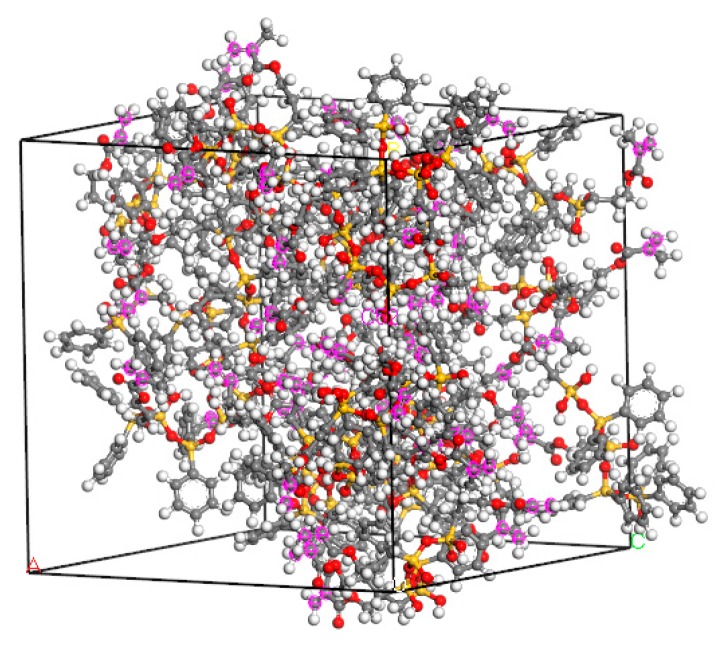
Molecular model of unpolymerized Ormocer photoresist.

A quench dynamics simulation was performed before the photoresist polymerization in order to bring the established Ormocer model closer to the actual photoresist structure. The parameters were: pressure: 0.1 GPa; temperature: 298 K; time step: 1 fs; total simulation time: 50 ps; number of steps: 1000; thermostat: Andersen; barostat: Berendsen. The final structure is shown in Figure 5, where the purple mark marks the C=C double bond to facilitate subsequent cross-linking reactions.

**Figure 6:**
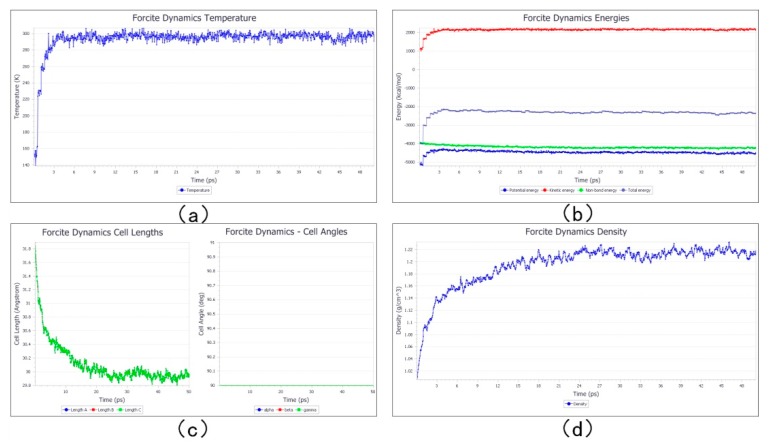
The quench dynamic simulation process before cross-linking changes with the simulation time. (**a**) The temperature variation with the simulation time. (**b**) The energy variation with the simulation time. (**c**) Structure size variation with the simulation time. (**d**) Density variation with the simulation time.

The stable structure of the photoresist was obtained after quench dynamic simulation. The change in the size density of the photoresist’s temperature and energy structure with the simulation time is shown in Figure 6. As can be seen from the figure, during the simulation, the temperature fluctuated around 298 K and remained stable; the energy tended to balance quickly. After 20 ps, the size and density of the structure also fluctuated slightly within a stable range.

### 3.3. Cross-Linking Simulation of Photoresist

**Figure 7:**
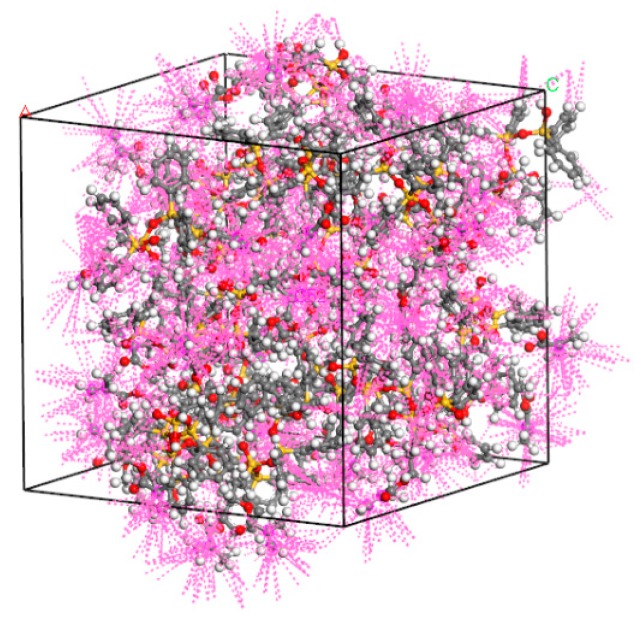
Unpolymerized photoresist molecular model reaction atom interaction degree.

To analyze the activity between adjacent carbon atoms, we used the Close Contact command in the Build toolbar of MS 2017. The active distance was set to 5 Å. As shown in Figure 7, the pink, dotted lines on the carbon atoms indicate the interactions among the atoms. The more dotted lines, the stronger the interaction, and the stronger carbon atoms are the cross-linked carbon atoms. By activity analysis, we can measure the carbon atoms that can cross-link.

**Figure 8:**
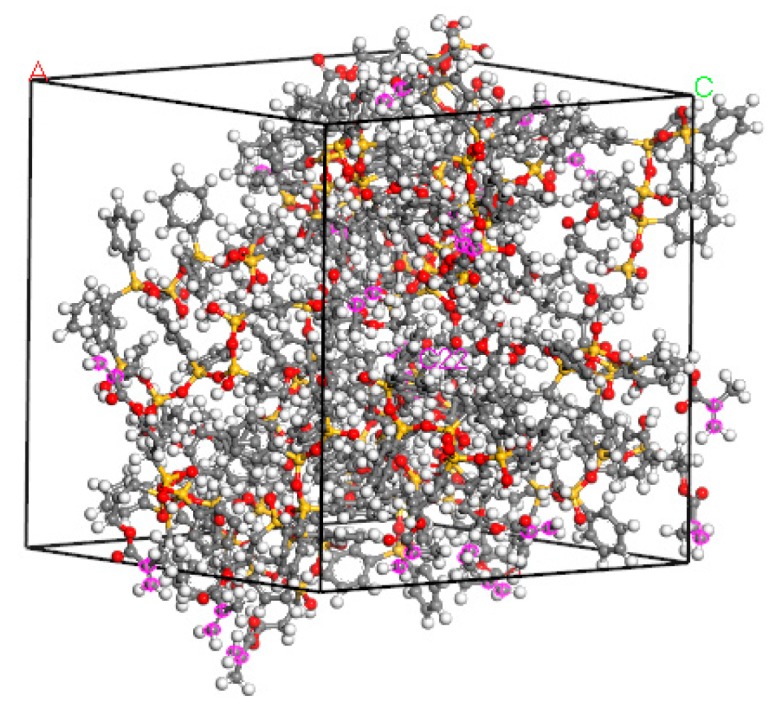
Photoresist structure model after polymerization

**Figure 9:**
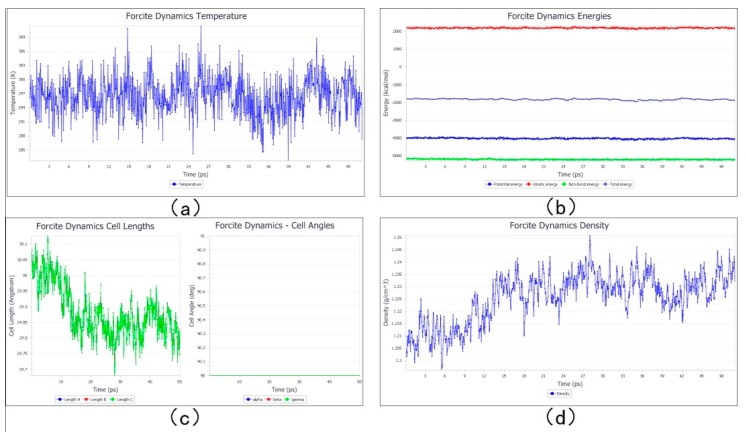
The quench dynamic simulation process after cross-linking changes with the simulation time. (**a**) The temperature variation with the simulation time. (**b**) The energy variation with the simulation time. (**c**) Structure size variation with the simulation time. (**d**) Density variation with the simulation time.

According to the cross-linking principle in Section 2.1, manual cross-linking was carried out to obtain the micro/nano structure after cross-linking. After completion of cross-linking, a 50 ps dynamics simulation was conducted to obtain a stable cross-linked structure, and the final structure is shown in Figure 8. From Figure 8, we can see that the purple marks are fewer, because after the cross-linking, we deleted the purple marks on the carbon atoms that were cross-linked. From the figure, we can see the cross-linking rate more intuitively. The changes in temperature, energy, structure size, and density with time in the final quench dynamic simulation are shown in Figure 9.

### 3.4. Contractility Analysis before and after Polymerization

Stable structures of the Ormocer molecular model before and after polymerization were obtained by quench dynamic simulation. The changes in the structure parameters over time before and after polymerization were compared. Figure 6a and Figure 9a show the dynamics simulation before and after the polymerization progression over time, and that temperature had a small fluctuation in the range of 298 ± 5 K (that is, the normal temperature was 25 °C). This shows that the temperature was constant during the kinetic simulation. It can be seen from Figure 6b that the energy was unstable at the beginning of the simulation. After a period of time, the simulated kinetic energy, potential energy, non-bond energy, and total energy reached a steady state, indicating that the energy was optimized and the structure was more stable. The energy shown in Figure 9b remained in a stable state, because the energy minimization optimization and molecular dynamics simulation were performed for every step of the cross-linking process to ensure the structure reached a stable state. The kinetic simulation energy after cross-linking was always in a stable minimum state. It can be seen from the left graph of Figure 6c that the size changed a lot, because the density we provided at the time of encapsulation was not the actual density of the photoresist, and the density changed during the dynamic simulation. After dynamic simulation, the density of the model reached the actual photoresist density, and the structure reached a stable state which makes the established model closer to the actual photoresist structure. The angle of the right image of Figure 6c remained 90°, indicating that the structure of the package was stable and that there was no excessive deformation during the dynamics simulation. The size of the left image in Figure 9c also tended to be stable, indicating that the structure had reached a steady state after kinetic simulation, and the right image also shows that the model changed evenly after cross-linking. The density seen in Figure 6d eventually stabilized, because the density of the model reached the actual density of the photoresist after kinetic simulation. In Figure 9d, the density finally stabilized, but the density after polymerization was larger than that before the polymerization because the van der Waals force became a covalent bond and the distance became small during polymerization. Therefore, the volume generation contraction and density became larger, and the structure was more encrypted.

Comparing the changes in the density before and after polymerization, it was found that the kinetic reaction before and after the polymerization reached a stable value after 20 ps, and there was a small fluctuation around the fixed value. Therefore, it can be considered that the structure had reached stability in this process, so the movement track of the last 30 ps was used as the research object to analyze the contraction before and after photoresist polymerization. The data obtained are shown in Table 2.

The density of the photoresist after polymerization was large, indicating that the photoresist became more compact during the polymerization. The length, width, height, and volume of the structure before and after contraction during the polymerization can be seen in Table 2.

The change rate of the data before and after polymerization was analyzed. According to the change rate, it was found that, after polymerization, the length, width, and height of the structure became smaller, the volume also shrank, the density became larger, and the structure became more compact. The calculated shrinkage ratio of the available size was 1.1169%, and the German micro resist given by the Ormocer resist was within the range of 1–2%; thus, it met the requirements. In addition, according to the size comparison of the simulated structures, it was found that the corresponding side length shrinkage rate was 0.3737% which indicates that, in the process of polymerization, the van der Waals force among the molecules became covalent bonds, the bond distance became shorter, a condensation reaction was generated, and the contraction produced by this reaction was a uniform contraction. According to these characteristics, the size of the processed structure can be enlarged evenly to compensate for the shrinkage reaction during polymerization. In order to better verify the correctness of the model established in this paper and the simulation, we also repeated the above simulation on the structures of 25 and 30 monomers and obtained a volume shrinkage for 25 monomers of 1.0384% and a volume shrinkage for 30 monomers of 1.1418%. Both of them met the volume shrinkage of Ormocer photoresist given by specifications which further proves the validity of the model established in this paper (Figures S1–S4 and Tables S1–S2).

## 4. Conclusions

In this paper, a stochastic multi-molecule modeling method was proposed to establish a photoresist model which was closer to the actual photoresist structure. The Random Copolymer function was used to establish the Ormocer monomer in this paper which completely simulated the generation process of photoresist monomers and made the monomer model more consistent with reality. Due to the fact that many kinds of monomers can be synthesized in the process of photoresist into monomers, if the number of encapsulated monomers is too small, the established model is only a part of the photoresist and cannot accurately represent the photoresist. Moreover, if the position of the monomer molecules is random, the cross-linking process is too random, and the cross-linking is inaccurate. Compared with previous studies, this paper used a large number of randomly generated monomers to construct a photoresist model in the process of encapsulation which made up for the inaccuracy of the cross-linking process of photoresist.

Three models with different sizes were cross-linked and simulated to obtain stable structures, and the stability of the structures was analyzed. It was found that the volume shrinkage of Ormocer photoresist before and after polymerization was between 1–2%. Therefore, the size of Ormocer photoresist can be designed with an additional 0.3% to compensate for the shrinkage caused by the covalent bond becoming Van der Waals force during polymerization. This result is consistent with a 1–2% shrinkage of Ormocer photoresist given by the specification. The validity of the model established in this paper was further verified which provides a reference for subsequent photoresist modeling and analysis. In addition, it also provides a basis for compensating shrinkage caused by chemical reactions in the two-photon induced photopolymerization process and reduces shrinkage reaction in the polymerization process to a certain extent.

## Figures and Tables

**Table 1:** The precursor and the mole content of the model system.

System	Precursor	Contents
Ormocer-I	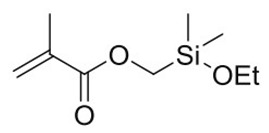	100%
Ormocer-II	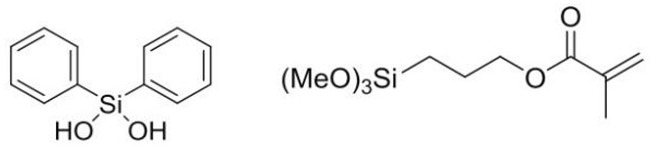	50%; 50%
Ormocer-III	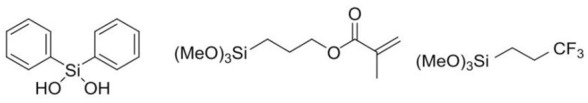	50%; 25%; 25%

**Table 2:** Changes in the Ormocer structure’s size before and after polymerization.

Trajectory	Data before Polymerization			Data after Polymerization		
	*ρ_bp_*	*L_bp_*	*V_bp_*	*ρ_ap_*	*L_ap_*	*V_ap_*
1	1.2090	29.9946	26,985.49	1.2364	29.7955	26,451.49
2	1.2022	30.0515	27,139.27	1.2365	29.7952	26,450.74
3	1.2028	30.0458	27,123.75	1.2257	29.8822	26,683.29
4	1.2163	29.9344	26,823.33	1.2346	29.8102	26,490.76
5	1.2164	29.9339	26,821.88	1.2331	29.8221	26,522.63
6	1.2159	29.9382	26,833.42	1.2323	29.8292	26,541.50
7	1.2165	29.9327	26,818.74	1.2315	29.8350	26,556.88
8	1.2156	29.9399	26,838.02	1.2482	29.7020	26,203.34
9	1.2197	29.9064	26,747.93	1.2288	29.8569	26,615.47
10	1.2132	29.9601	26,892.42	1.2278	29.8652	26,637.75
11	1.2173	29.9265	26,801.94	1.2431	29.7402	26,309.50
12	1.2079	30.0038	27,010.17	1.2358	29.8009	26,466.04
13	1.2017	30.0555	27,150.21	1.2332	29.8216	26,521.23
14	1.2069	30.0117	27,031.63	1.2217	29.9145	26,769.87
15	1.2137	29.9557	26,880.69	1.2391	29.7741	26,394.71
16	1.2079	30.0038	27,010.26	1.2254	29.8849	26,690.51
17	1.2134	29.9587	26,888.52	1.2344	29.8123	26,496.32
18	1.2194	29.9094	26,756.11	1.2248	29.8897	26,703.23
19	1.2189	29.9130	26,765.80	1.2233	29.9015	26,734.89
20	1.2127	29.9638	26,902.40	1.2257	29.8826	26,684.24
21	1.2234	29.8763	26,667.37	1.2234	29.9008	26,733.05
22	1.2253	29.8608	26,626.03	1.2179	29.9456	26,853.44
23	1.2083	30.0003	27,000.73	1.2337	29.8177	26,510.65
24	1.2226	29.8831	26,685.55	1.2295	29.8514	26,600.63
25	1.2155	29.9412	26,841.64	1.2312	29.8374	26,563.39
26	1.2310	29.8152	26,504.15	1.2257	29.8822	26,683.05
27	1.2221	29.8870	26,696.09	1.2317	29.8334	26,552.70
28	1.2193	29.9099	26,757.51	1.2399	29.7677	26,377.64
29	1.2048	30.0294	27,079.43	1.2307	29.8414	26,574.07
30	1.2178	29.9219	26,789.78	1.2344	29.8122	26,496.14
Average	1.2146	29.9488	26,862.34	1.2313	29.8369	26,562.31

Note: *ρ* represents the density of the structural model in g/cm^3^. L represents the side length of the photoresist structure before and after the polymerization, and the unit is Å. V represents the volume of the package structure, and the unit is Å3.

## Supplementary Materials

The following are available online at https://www.mdpi.com/1996-1944/12/23/3876/s1, Figure S1: The quench dynamic simulation process before crosslinking changes with the simulation time (**a**) The temperature varies with the simulation time (**b**) The energy varies with the simulation time (**c**) Structure size varies with simulation time (**d**) Density varies with simulation time, Figure S2: The quench dynamic simulation process after crosslinking changes with the simulation time (**a**) The temperature varies with the simulation time (**b**) The energy varies with the simulation time (**c**) Structure size varies with simulation time (**d**) Density varies with simulation time, Figure S3: The quench dynamic simulation process before crosslinking changes with the simulation time (**a**) The temperature varies with the simulation time (**b**) The energy varies with the simulation time (**c**) Structure size varies with simulation time (**d**) Density varies with simulation time, Figure S4: The quench dynamic simulation process after crosslinking changes with the simulation time (**a**) The temperature varies with the simulation time (**b**) The energy varies with the simulation time (**c**) Structure size varies with simulation time (**d**) Density varies with simulation time, Table S1: Changes of Ormocer structure size before and after polymerization, Table S2: Changes of Ormocer structure size before and after polymerization.
