# Supplementary material for: Stochastic Multi-Molecular Modeling Method of Organic-Modified Ceramics in Two-Photon Induced Photopolymerization
Source: Materials (Basel). 2019 Nov 24;12(23):3876. doi: 10.3390/ma12233876 (PMC6926505; doi:10.3390/ma12233876)
Supplement: Supplementary file 1 [file materials-12-03876-s001.pdf]

materials

Supplementary Materials: Stochastic Multi-molecular Modeling Method of Organic Modified ceramics in Two-Photon induced Photopolymerization

Jieqiong Lin, Peng Liu, Xian Jing, Mingming Lu, Kaixuan Wang and Jie Sun

1. Molecular Dynamics Simulation of 25 Ormocer Photoresist Monomer Models

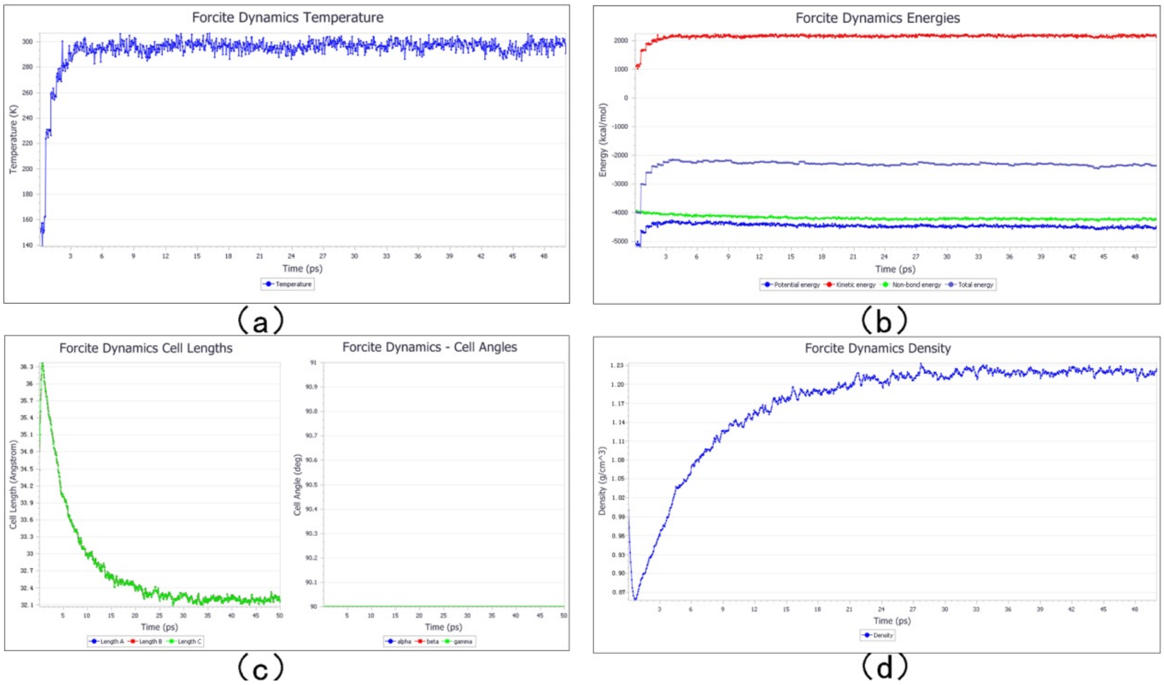

Figure S1. The quench dynamic simulation process before cross-linking changes with the simulation time. (**a**) The temperature variation with the simulation time. (**b**) The energy variation with the simulation time. (**c**) Structure size variation with the simulation time. (**d**) Density variation with the simulation time.

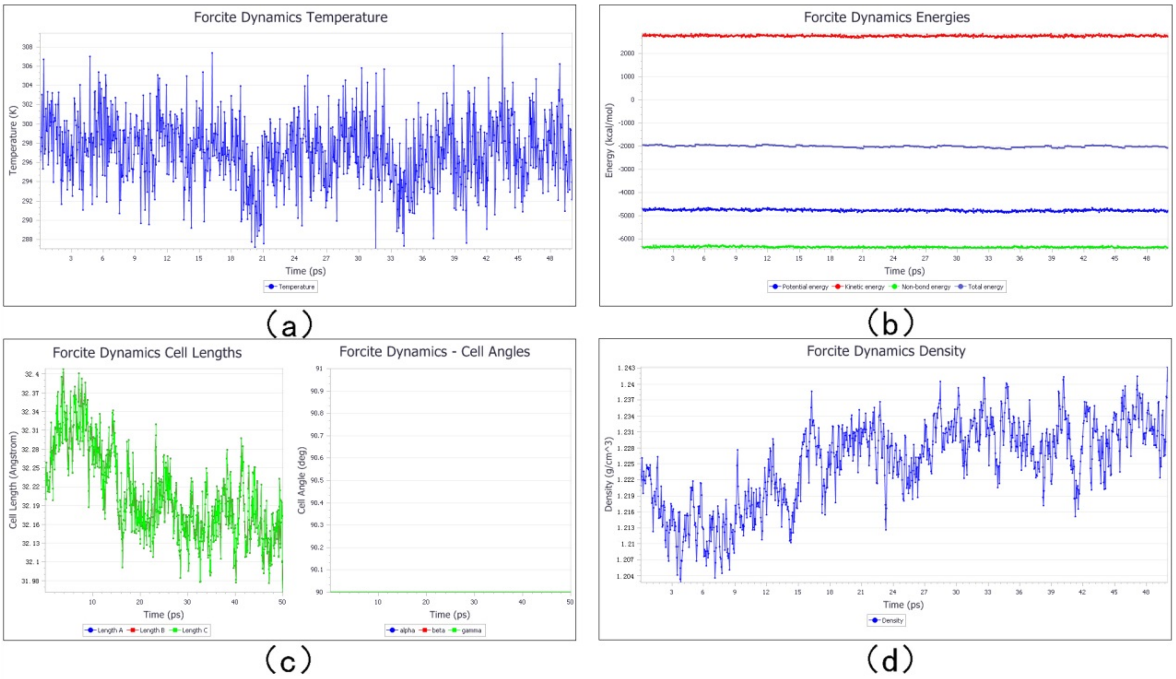

Figure S2. The quench dynamic simulation process after cross-linking changes with the simulation time. (**a**) The temperature variation with the simulation time. (**b**) The energy variation with the simulation time. (**c**) Structure size variation with the simulation time. (**d**) Density variation with the simulation time.

Table S1. Changes of Ormocer structure size before and after polymerization.

Trajectory	Data before Polymerization			Data after Polymerization		
	ρ_{bp}	L_{bp}	V_{bp}	ρ_{ap}	L_{ap}	V_{ap}
1	1.2094	32.3160	33748.38	1.2299	32.1651	33277.86
2	1.2060	32.3463	33843.53	1.2299	32.1651	33277.86
3	1.2025	32.3777	33942.03	1.2305	32.1608	33264.33
4	1.1998	32.4019	34018.41	1.2233	32.2237	33460.18
5	1.2050	32.3553	33871.61	1.2156	32.2917	33672.19
6	1.1997	32.4029	34021.25	1.2235	32.2220	33454.71
7	1.2225	32.2002	33386.74	1.2312	32.1547	33245.41
8	1.2122	32.2911	33670.43	1.2306	32.1599	33261.62
9	1.2070	32.3374	33815.49	1.2321	32.1468	33221.12
10	1.2202	32.2204	33449.68	1.2334	32.1355	33186.09
11	1.2245	32.1826	33332.21	1.2238	32.2194	33446.50
12	1.2159	32.2583	33567.97	1.2286	32.1773	33315.79
13	1.2257	32.1821	33299.58	1.2318	32.1494	33229.21
14	1.2148	32.2680	33598.37	1.2250	32.2088	33413.72
15	1.2116	32.2964	33687.10	1.2335	32.1347	33183.40
16	1.2114	32.2982	33692.66	1.2332	32.1373	33191.48
17	1.2204	32.2184	33444.19	1.2332	32.1373	33191.48
18	1.2125	32.2884	33662.10	1.2274	32.1878	33348.37
19	1.2127	32.2867	33656.55	1.2198	32.2546	33556.22

20	1.2144	32.2716	33609.43	1.2326	32.1425	33207.64
21	1.1975	32.4227	34083.75	1.2265	32.1948	33370.13
22	1.2187	32.2336	33490.85	1.2218	32.2370	33501.27
23	1.2241	32.1860	33343.11	1.2311	32.1573	33253.51
24	1.2166	32.2521	33548.66	1.2209	32.2440	33523.23
25	1.2179	32.2406	33512.85	1.2350	32.1216	33143.08
26	1.2117	32.2955	33684.32	1.2403	32.0758	33001.41
27	1.2151	32.2654	33590.07	1.2339	32.1312	33172.64
28	1.2215	32.2089	33414.08	1.2298	32.1669	33283.27
29	1.2037	32.3696	33916.55	1.2312	32.1547	33245.41
30	1.2185	32.2380	33504.59	1.2410	32.0698	32982.79
Average	1.2131	32.2831	33645.45	1.2293	32.1677	33296.06

Note: In the table, ρ represents the density of the structural model in *g/cm3*. *L* represents the side length of the photoresist structure before and after the polymerization, and the unit is $\AA$. *V* represents the volume of the package structure, and the unit is $\AA^3$. Volume shrinkage is 1.0384%.

2. Molecular Dynamics Simulation of 30 Ormocer Photoresist Monomer Models

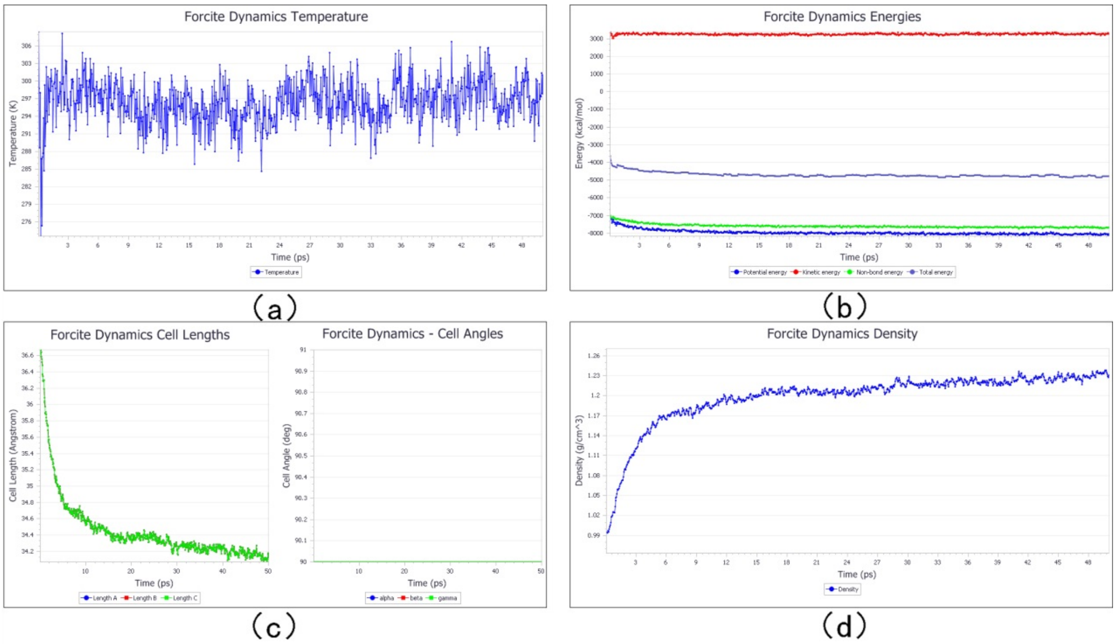

Figure S3. The quench dynamic simulation process before cross-linking changes with the simulation time. (**a**) The temperature variation with the simulation time. (**b**) The energy variation with the simulation time. (**c**) Structure size variation with the simulation time. (**d**) Density variation with the simulation time.

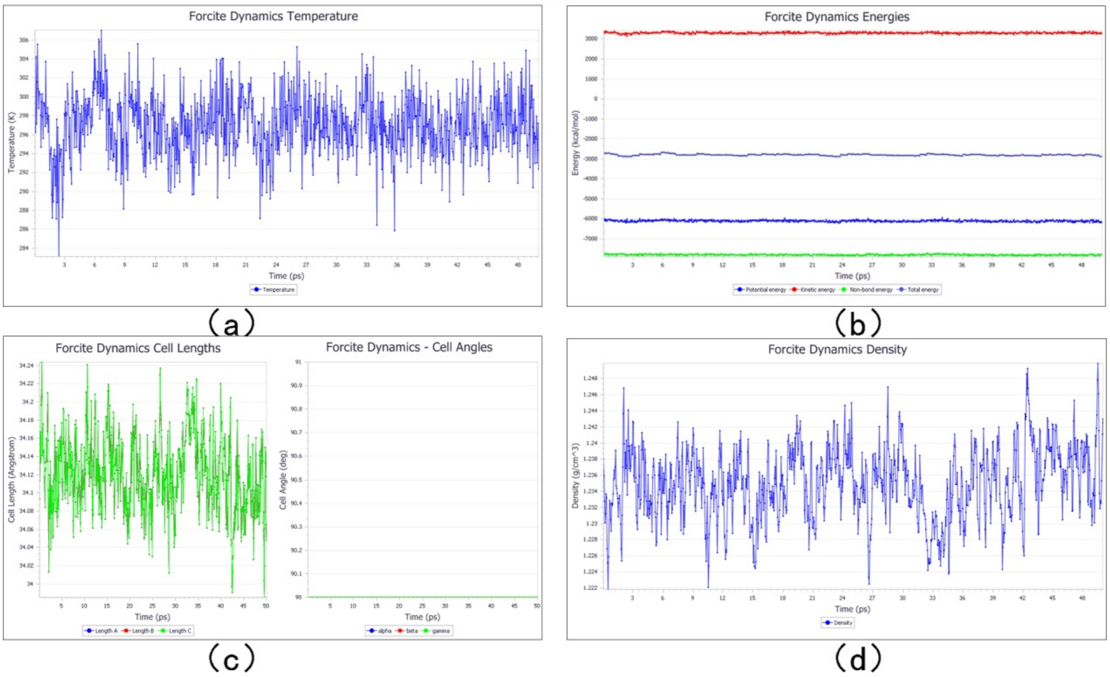

Figure S4. The quench dynamic simulation process after cross-linking changes with the simulation time. (**a**) The temperature variation with the simulation time. (**b**) The energy variation with the simulation time. (**c**) Structure size variation with the simulation time. (**d**) Density variation with the simulation time.

Table S2. Changes of Ormocer structure size before and after polymerization.

**Trajectory**	**Data before Polymerization**			**Data after Polymerization**		
	ρ_{bp}	L_{bp}	V_{bp}	ρ_{ap}	L_{ap}	V_{ap}
1	1.2043	34.3928	40681.90	1.2300	34.1671	39866.37
2	1.2057	34.3794	40634.66	1.2354	34.1174	39712.68
3	1.2026	34.4090	40739.41	1.2390	34.0848	39598.80
4	1.2023	34.4118	40749.57	1.2349	34.1221	39729.08
5	1.1995	34.4386	40844.69	1.2333	34.1369	39780.75
6	1.2111	34.3283	40453.48	1.2337	34.1331	39767.33
7	1.2067	34.3699	40600.99	1.2406	34.0701	39547.53
8	1.2053	34.3832	40648.15	1.2340	34.1313	39760.94
9	1.2190	34.2539	40191.31	1.2346	34.1253	39740.11
10	1.2177	34.2661	40234.22	1.2367	34.1055	39671.11
11	1.2109	34.3302	40460.16	1.2354	34.1177	39713.76
12	1.2114	34.3254	40443.46	1.2324	34.1453	39810.12
13	1.2177	34.2661	40234.22	1.2318	34.1503	39827.80
14	1.2134	34.3066	40376.80	1.2290	34.1769	39920.54
15	1.2204	34.2408	40145.21	1.2332	34.1376	39783.01
16	1.2126	34.3141	40403.44	1.2325	34.1443	39806.56
17	1.2137	34.3037	40366.82	1.2341	34.1301	39756.74
18	1.2117	34.3226	40433.45	1.2378	34.0953	39635.49
19	1.2173	34.2699	40247.44	1.2409	34.0665	39535.19

20	1.2136	34.3047	40370.15	1.2288	34.1783	39925.74
21	1.2338	34.1164	39709.20	1.2348	34.1231	39732.51
22	1.2151	34.2905	40320.31	1.2294	34.1728	39906.56
23	1.2204	34.2408	40145.21	1.2404	34.0717	39553.31
24	1.2205	34.2399	40141.92	1.2345	34.1259	39742.24
25	1.2322	34.1312	39760.76	1.2424	34.0537	39490.62
26	1.2269	34.1803	39932.52	1.2371	34.1022	39659.52
27	1.2273	34.1766	39919.51	1.2355	34.1172	39711.97
28	1.2240	34.2072	40027.13	1.2367	34.1061	39673.18
29	1.2314	34.1386	39786.59	1.2325	34.1440	39805.61
30	1.2262	34.1868	39955.32	1.2425	34.0526	39486.73
Average	1.2159	34.2453	40293.78	1.2351	34.1202	39722.40

Note: In the table, *ρ* represents the density of the structural model in g/cm^3. *L* represents the side length of the photoresist structure before and after the polymerization, and the unit is *Å*. *V* represents the volume of the package structure, and the unit is $Å^3$. Volume shrinkage is 1.4180%.
